# Identification of a sixteen-microRNA signature as prognostic biomarker for stage II and III colon cancer

**DOI:** 10.18632/oncotarget.21237

**Published:** 2017-09-23

**Authors:** Havjin Jacob, Luka Stanisavljevic, Kristian Eeg Storli, Kjersti E Hestetun, Olav Dahl, Mette P Myklebust

**Affiliations:** ^1^ Department of Clinical Science, Faculty of Medicine, University of Bergen, Bergen, Norway; ^2^ Department of Oncology and Medical Physics, Haukeland University Hospital, Bergen, Norway; ^3^ Department of Surgery, Haraldsplass Deaconess Hospital, Bergen, Norway

**Keywords:** miRNA, signature, colon cancer, recurrence, RT-qPCR

## Abstract

Despite advances in colon cancer research and novel therapies, high risk of recurrence remains a major challenge. This study reports miRNA expression profiling as a biomarker for the prognosis of TNM stage II and III colon cancer.

Fresh frozen biopsies from the study cohort (N=111) were analyzed for miRNA by RT-qPCR and LASSO regression analysis was used to build a classifier of miRNAs. The prognostic accuracy was tested and the classifier was validated in an independent colon cohort (TCGA-COAD, N=209).

The LASSO regression analysis identified a 16-miRNA signature including miR-143-5p, miR-27a-3p, miR-31-5p, miR-181a-5p, miR-30b-5p, miR-30d-5p, miR-146a-5p, miR-23a-3p, miR-150-5p, miR-210-3p, miR-25-3p, miR-196a-5p, miR-148a-3p, miR-222-3p, miR-30c-5p and miR-223-3p. A low 16-miRNA signature was associated with better 5-year disease-free survival (DFS) in the study cohort than a high signature (93 % versus 58 %; p< 0.001). The signature was an independent prognostic factor for better 5-year DFS in multivariate analyses (HR 21.4; 95% CI: 4.21-108.7; p< 0.001). The results in the validation cohort were consistent with the study cohort in univariate (77 % versus 65 %; p= 0.045) and multivariate analyses (HR 2.0; 95% CI: 1.04-3.89; p=0.039).

We identified a 16-miRNA signature as a reliable prognostic biomarker for classification of colon cancer stage II and III patients into groups with low and high risk for recurrence.

## INTRODUCTION

Colon cancer is the fourth most common cause of cancer-related deaths, with increasing incidence and high mortality worldwide [1, 2]. The prognosis of colon cancer is predominantly based on the tumor stage and clinicopathological features. However, the heterogeneity of the disease makes it difficult to determine patient prognosis based on these traditional factors. The transformation of normal mucosa into tumor is driven by a series of genetic and epigenetic changes [3, 4]. Therefore understanding the molecular mechanisms of colon cancer is crucial to improve diagnosis and treatment. One of the important pathways that lead to colon cancer is chromosomal instability characterized by mutations in particular genes. Among the most common mutated genes in colon cancer KRAS (Exon 2 codon 13) and BRAF are associated with poor prognosis [5–7]. However, the use of these biomarkers is part of standard clinical practice in metastatic patients. Moreover, mutations in the TP53 gene are found to be associated with poor survival but further studies including more extensive cohorts are needed to consider whether TP53 is a useful prognostic marker [5, 8]. Microsatellite instability (MSI) is presented as a biomarker for stage II colon cancer, but it cannot predict whether patients are at high or low risk of recurrence in other stages [9–12]. Further, the recently published “consensus molecular subtyping” classification enables the categorization of tumors into four subtypes [13, 14]. However, little is known about the prognostic and predictive reliability of this classification in clinical use. Thus, establishing novel biomarkers to improve classification of patients and adding information to the TNM staging system is highly desirable.

Lately, there have been an increasing number of microRNA (miRNA) studies in several cancers including colorectal cancer [15]. MiRNAs are conserved, small non-coding molecules of RNA which were first described in 1993 [16]. The main function of miRNAs is regulation of gene expression post-transcriptionally to repress translation and/or promote mRNA degradation [17]. More than 30% of the genome is predicted to be regulated by miRNAs and different studies have shown the involvement of miRNA in cellular processes such as proliferation, differentiation, metabolism and organogenesis [17, 18].

Further, dysregulation of miRNAs is associated with a number of cancers, including colon cancer by acting as tumor suppressors or onco-miRNAs [15, 19–21]. Currently, studies are focusing on miRNA signatures and their association with cancer stage, progression, prognosis and treatment response. The prognostic value of miRNA signatures has been identified in colon and other cancers in several previous studies [21–25], but no consensus regarding miRNAs in colon cancer has been reached.

Here, we performed miRNA profiling to identify a panel of miRNA signature using LASSO- analysis to predict recurrence in TNM stage II and III colon cancer patients.

## RESULTS

A total of 111 TNM stage II and III colon cancer patients with a mean age of 72.5 years (range 23-93) were included in the study cohort (Table 1). Sixty six patients were classified as TNM stage II and 45 as stage III patients. Among stage II patients there were nine recurrences, in stage III the number of recurrence was 19. Eighty eight patients had well or moderately differentiated tumors and 21 patients had poor tumor differentiation. Further, 101 patients had adenocarcinoma whereas 10 patients had signet ring and mucinous carcinomas. The validation cohort, TCGA-COAD, included 209 TNM stage II and III colon cancer patients with a mean age of 66.5 years. The number of TNM stage II patients was 122 (23 with recurrence) and TNM stage III was 87 patients (19 with recurrence).

**Table 1:** The clinicopathological variables and their association with low- and high 16-miRNA signature

Study Cohort n= 111	Total (nL)	16-miRNA signature		*p*-value^a^
		Low (%) n= 48	High (%) n= 63	
Age				NS
≤72.5	49	47.9	41.3	
>72.5	62	52.1	58.7	
Gender				NS
Female	51	50	42.9	
Male	60	50	57.1	
TNM stage^b^				0.004
II	66	75	47.6	
III	45	25	52.4	
MMR status				NS
Deficient	24	22.7	26.4	
Proficient	73	77.3	73.6	
ND	14			
Tumor differentiation				NS
Well/Moderate	88	81.3	77.8	
Poor	21	18.8	19	
ND	2	0	3.2	
Histology type				NS
Adenocarcinoma	101	89.6	92.1	
Variant^c^	10	10.4	7.9	
Adjuvant therapy				NS
Yes	25	20.8	23.8	
No	86	79.2	76.2	
**Validation Cohort n= 209**		**Low (%) n= 90**	**High (%) n= 119**	
Age				NS
≤66.5	92	37.8	48.7	
>66.5	117	62.2	51.3	
Gender				NS
Female	98	41.1	51.3	
Male	111	58.9	48.7	
TNM stage				NS
II	122	61.1	56.3	
III	87	38.9	43.7	
MSI status				NS
MSS	135	58.9	68.9	
MSI Low	30	14.4	14.3	
MSI High	44	26.7	16.8	

^a^*P*-values from Chi-square, ^b^TNM tumour, node metastasis classification of malignant tumours, ^c^Variant includes signet ring and mucinous carcinoma, NS= not significant (p > 0.05), ND= not determined.

The LASSO approach identified a signature of 16 miRNAs as the best predictor of recurrence (Supplementary Figure 1). The miRNAs were: miR-143-5p, miR-27a-3p, miR-31-5p, miR-181a-5p, miR-30b-5p, miR-30d-5p, miR-146a-5p, miR-23a-3p, miR-150-5p, miR-210-3p, miR-25-3p, miR-196a-5p, miR-148a-3p, miR-222-3p, miR-30c-5p and miR-223-3p. The Prognostic index (PI)s were calculated from dichotomized expression of each miRNA in the signature and the coefficients using the formula PI = (miR-143-5p × 0.028) + (miR-27a-3p × (−0.065)) + (miR-31-5p × 0.069) + (miR-181a-5p × 0.134) + (miR-30b-5p × 1.836) + (miR-30d-5p × 0.212) + (miR-146a-5p × (−0.020)) + (miR-23a-3p × (−0.619)) + (miR-150-5p × 0.142) + (miR-210-3p × 0.044) + (miR-25-3p × (−0.091)) + (miR-196a-5p × 0.014) + (miR-148a-3p × (−0.144)) + (miR-222-3p × 0.060) + (miR-30c-5p × (−1.045)) + (miR-223-3p × (−0.164)).

**Figure 1:**
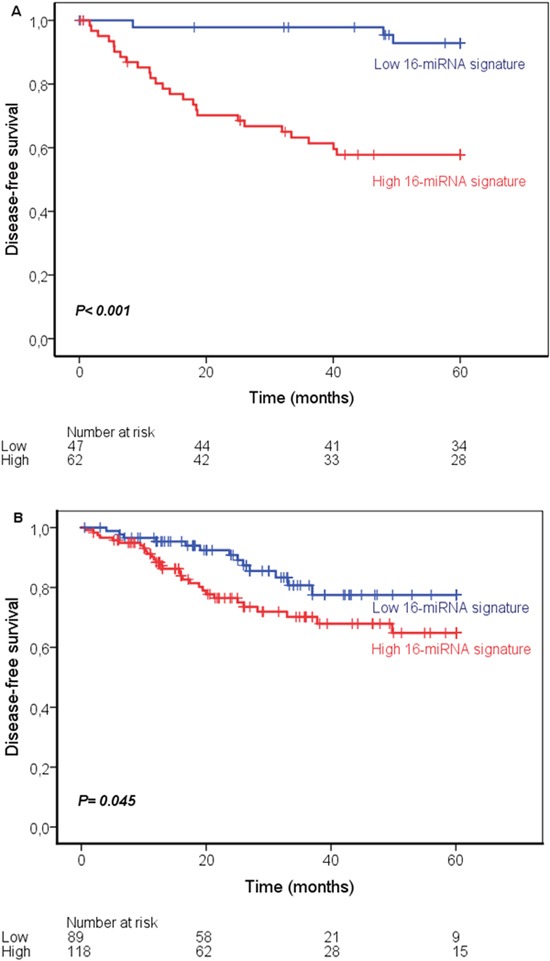
Kaplan-Meier curves showing disease-free survival in stage II and III colon cancer according to the 16-miRNA signature in the study cohort **(A)** and the validation cohort **(B)**.

The cut-point value of the PI was assessed using ROC-curve and this divided the patients into low and high risk of recurrence. Kaplan-Meier analysis with the log-rank test was further used to evaluate the ability of the 16-miRNA signature to predict survival in TNM stage II and III colon cancer patients. Patients with a low 16-miRNA signature showed better 5-year DFS, than patients with high signature, 93% versus 58%, respectively (p< 0.001; Figure 1A). The associations of the clinicopathological variables with the 16-miRNA signature are shown in Table 1. There was a statistically significant association between TNM stage and the 16-miRNA signature (p= 0.004). The variables, age, gender, differentiation, MMR-status, tumor differentiation, histology type and adjuvant therapy were not significantly associated with the 16-miRNA signature.

When the signature together with the clinicopathological features age, gender, TNM stage, differentiation and MMR-status were entered in multivariate Cox regression analysis, the signature remained as an independent prognostic factor (HR 21.4; 95% CI: 4.21-108.7; p< 0.001, Table 2).

**Table 2:** Univariate and multivariate analyses for the 16-miRNA signature in the study- and the validation cohort

	Univariate analyses			Multivariate analyses		
	HR	95% CI	p-value	HR	95% CI	p-value
**Study cohort**						
Age (mean)			NS			NS
≤72.5	1			1		
>72.5	0.83	(0.39-1.75)		0.70	(0.29-1.66)	
Gender			NS			NS
Female	1			1		
Male	1.17	(0.55-2.47)		0.70	(0.29-1.66)	
TNM^a^ stage			0.001			NS
II	1			1		
III	3.71	(1.68-8.22)		2.06	(0.79-5.33)	
Differentiation			0.010			0.007
Well/Moderate	1			1		
Poor	2.78	(1.28-6.03)		7.71	(2.16-27.4)	
MMR-status			NS			0.059
Proficient	1			1		
Deficient	0.48	(0.14-1.65)		0.24	(0.05-1.05)	
16-miRNA signature			0.001			**<0.001**
Low	1			1		
High	8.20	(2.47-27.2)		21.4	(4.21-108.7)	
**Validation cohort**						
Age (mean)			NS			NS
≤66.5	1			1		
>66.5	0.82	(0.44-1.52)		0.84	(0.44-1.57)	
Gender			NS			NS
Female	1			1		
Male	1.56	(0.83-2.91)		1.72	(0.90-3.27)	
TNM^a^ stage			NS			NS
(II)	1			1		
(III)	1.38	(0.75-2.54)		1.45	(0.77-2.70)	
MSI-status						
MSS	1		NS	1		NS
MSI Low	1.59	(0.74-3.41)		1.88	(0.87-4.07)	
MSI High	0.79	(0.34-1.82)		0.92	(0.39-2.14)	
16-miRNA signature			0.049			**0.039**
Low	1			1		
High	1.93	(1.00-3.72)		2.00	(1.04-3.89)	

HR=hazard ratio, CI=confidence interval, NS= not significant (p > 0.05). Differentiation not available in the validation cohort, ^a^TNM tumour, node, metastasis classification of malignant tumours.

These results show that TNM and tumor differentiation are statistically significant prognostic factors in univariate analyses (HR 3.71; 95% CI: 1.68-8.22; p=0.001), (HR 2.78; 95% CI: 1.28-6.03; p=0.010), respectively. Differentiation remained statistically significant when entered in the multivariate analyses with the 16-miRNA signature (HR 2.06; 95% CI: 0.79-5.33; p=0.007) but TNM stage did not (Table 2).

The validity of the 16-miRNA signature was evaluated using the independent publically available TCGA-COAD dataset. The Kaplan-Meier survival curve with the log-rank test confirmed the robustness of the prognostic value of the 16-miRNA signature in TCGA-COAD. A low 16-miRNA signature was associated with better 5-years DFS, 77 % versus 65 % in low versus high 16-miRNA signature (p= 0.045; Figure 1B). The 16-miRNA signature and the variables age, gender, TNM stage and MSI status (information regarding tumor differentiation was not available) were entered in the multivariate analyses. No statistically significant associations were found between the signature and the variables age, gender, TNM stage and MSI-status in the TCGA-COAD (Table 1). The signature remained a statistically significant independent prognostic variable in the multivariate analyses (HR 2.00; 95% CI: 1.03-3.89; p=0.039) in TCGA data (Table 2).

## DISCUSSION

In this study, we present the development and validation of a 16-miRNA signature associated with recurrence in TNM stage II and III colon cancer patients. The prognostic value of the signature was demonstrated to be a strong classifier in the univariate analyses, and it remained a strong prognostic factor in multivariate analyses. This implies that a low signature is an independent prognostic factor for better survival.

The results of the validation cohort are consistent with the study cohort in both univariate and multivariate analyses. However, the signature appears to be a stronger prognostic marker in the study cohort than in the validation data. The patients included in the TCGA data have a lower average age than the study cohort (66.5 versus 72.5 years; Table 1) which may have an impact on the differences. Also the survival rates in the two cohorts are a bit different. The 5-year DFS for stage II colon cancer patients available for analysis in TCGA-COAD is lower than in our study cohort (72% versus 85%, respectively). The 5-year DFS rates for stage II patients in TCGA-COAD are lower than expected (80-84 %) [26]. For stage III patients the 5-year DFS is better in the TCGA-cohort than in our study cohort (69% versus 56%, respectively). The expected 5-year DFS for stage III patients is 59-66% [26]. Further, MMR status by immunohistochemistry was available for only a limited number of patients in the TCGA-COAD cohort, therefore MSI-status was used in the multivariate analyses (Table 2). These differences may contribute to the performance of the 16-miRNA signature as a prognostic marker in the TCGA-COAD data compared to the study cohort. Several studies have identified miRNA-signatures in cancer, highlighting the role of miRNAs in cancer progression and suggesting that miRNA-signatures may have a prognostic value in cancer [22, 27–29]. The results are inconsistent, possibly because of the small numbers of patients in the study cohorts, lack of a validation cohort or varying methodology used in different studies to build a classifier based on a miRNA panel. A previous study identified a six-miRNA classifier that included only one of the miRNAs in our signature (miR-143) [22]. An explanation for this might be that our study included stage II and III colon cancer patients, whereas the mentioned study included only stage II colon cancer patients. Three of the miRNAs in our signature (miR-150, miR-223 and miR-23a) are previously identified as an exosomal miRNA signature in serum with potential as a biomarker of colon cancer [30, 31]. In addition, miR-23a-3p and miR-27a-3p is presented in another study as diagnostic and prognostic biomarkers in a serum-based miRNA signature in colon cancer [32]. Thus, four of the miRNAs in our signature is also documented in circulating miRNA-signatures in colon cancer.

Further, a panel of three miRNAs (miR-146a, miR-222 and miR-223) is identified as a biomarker for early detection in lung cancer [33]. The expression of colon cancer associated miRNAs in serum indicates that the tumor expresses high levels of the specific miRNA, thus these results published by others support our finding of these miRNAs as prognostic biomarkers in colon cancer.

The biological functions of the miRNAs in our signature in colon cancer are partly studied. For instance, miR-31, miR-143, miR-196a and miR-223 are involved in the regulation of RAS-MAPK and PI3K-AKT cascades [34–37]. High expression of miR-148a is found to be associated with progression in colorectal cancer [38] and miR-30c is involved with ADAM19 in colon cancer cell lines [39]. Another study showed that hypoxia induced miR-210 has an important role in the regulation of colon cancer [40]. Further, miR-25 is downregulated in colon cancer and putatively targets Smad7 as a tumor suppressor [41]. Based on these findings, the miRNAs included in our signature are involved in several crucial regulatory pathways in colon cancer.

Surgery is the primary treatment of colon cancer followed by adjuvant chemotherapy for stage III and high-risk TNM stage II patients [42]. Adjuvant chemotherapy has non-negligible side effects, and it is still debated whether high-risk stage II patients benefit from it. Moreover, many stage III patients do not gain from adjuvant chemotherapy as they are cured by surgery alone [43, 44]. Therefore, identifying a reliable prognostic biomarker that stratifies patients into risk groups for recurrence is highly desirable by the clinicians to administrate chemotherapy. Our results suggest that the 16-miRNA signature adds valuable information to the clinical features. Using our signature, the oncologists can identify the patients with high risk for recurrence to whom adjuvant chemotherapy should be considered.

Integration of the 16 miRNAs into one tool using the LASSO Cox regression method was shown to have a great prognostic accuracy in our study cohort and the validation data set. Thus, designing a ready-to-use panel containing the 16-miRNAs enables the laboratory to accomplish the miRNA expression profiling fast and at a reasonable cost by RT-qPCR. Further, as the coefficients or “weights” for each miRNA are constants, the PI for each individual patient can be quickly calculated using the miRNA expression levels retrieved from the RT-qPCR analysis. As for all laboratory analyses used for decision making in clinical care, each laboratory must thoroughly establish the steps of the analysis.

In summary, we present identification of a novel classifier based on 16-miRNAs with potential as a prognostic biomarker to stratify colon cancer stage II and III patients to low- and high risk for colon cancer recurrence.

## MATERIALS AND METHODS

### Patient cohorts

**Figure 2:**
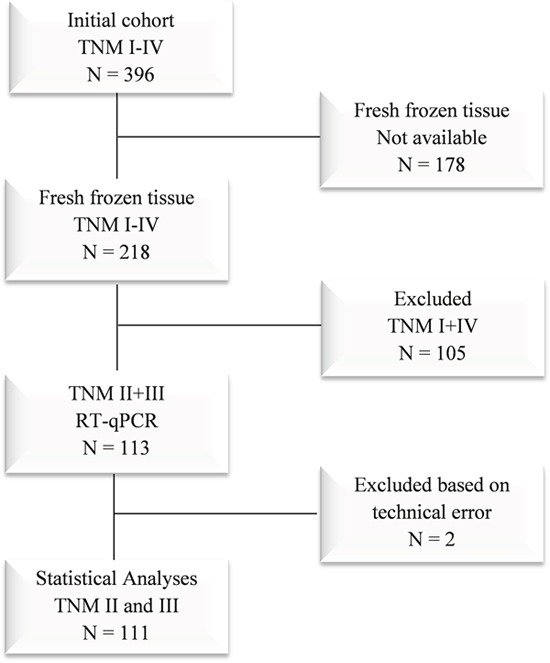
Flowchart of the study cohort

The study cohort includes patients enrolled in a prospective study from January 2007 to December 2011 where 396 consecutive patients at Haraldsplass Deaconess Hospital had surgery for primary TNM stage I to IV colon cancer [45]. In the present study, we have focused on the TNM stage II and III patients of this cohort with available fresh frozen biopsies (N=111). The flowchart of the study is presented in Figure 2. Distant metastases were excluded by chest and abdominal CT imaging. When technically feasible, a complete mesocolic resection (CME) with high vascular tie was performed [46]. None of the patients received radiotherapy. TNM stage II and III patients received adjuvant chemotherapy, according to the national clinical guidelines [47]. Patients less than 70 years of age were treated with the Nordic FLOX regimen, while patients from 70 to 75 years of age were treated with Nordic FLv [48, 49]. The validation cohort includes 211 TNM stage II and III patients with available adequate survival information from the TCGA-COAD cohort (The Cancer Genome Atlas; https://cancergenome.nih.gov/; Supplementary Figure 2).

### RNA extraction

Tissue samples were collected in RNA*later*™ immediately after resection and stored frozen at −80 °C. Total RNA was extracted from colon cancer tissue samples and ten adjacent normal colon tissues using miRNeasy, Mini kit (QIAGEN) according to the manufacturer's protocol. Briefly, 30 mg tissue sample was lyzed in 700 μl QIAzol Lysis Reagent using Tissuelyzer (QIAGEN) for 10 minutes at 25 HZ. Chloroform (140 μl) was added to the homogenate, incubated for 5 minutes and centrifuged for 15 minutes to separate RNA. Next, on-column washing steps were performed, one time with 700 μl RWT buffer and two times with 500 μl RPE buffer. Finally, the RNA was eluted in 40 μl RNase-free water. Total RNA was quantified using a NanoDrop spectrophotometer and the quality of the RNA was assessed using Agilent RNA Bioanalyzer (Agilent RNA 6000 Nano Assay protocol-edition April 2007). The RNA samples were stored at -80 °C until further processing.

### MicroRNA quantification by RT-qPCR

The expression of the miRNA profile was assessed using the miRCURY LNA Universal RT miRNA PCR (Exiqon, Denmark). Briefly, an input of 5 ng/ μl total RNA was used in universal cDNA synthesis and qPCR performed using miRNA specific LNA enhanced qPCR primers in Pick and Mix panels and ExiLENT SYBR® Green master mix (Exiqon, v6.0, 2014) as recommended by the manufacturer. The Pick and Mix panels included a selection of 84 miRNAs that are linked to cancer (Supplementary Figure 3). Further, the panels contained inter-plate calibrators (UniSp3) and RNA spike-in controls (cel-miR-39-3p, UniSp2, UniSp4, UniSp5 and UniSp6) for quality control purpose. A robot was used for pipetting, and the RT-qPCR was analyzed on LightCycler 480.

### Ethics

The study protocol was approved by the Regional Committee for Medical Research Ethics of Western Norway and the Data Inspectorate for National Registries. The preceding clinical trial is also registered at the U.S National Institute of Health (ClinicalTrials.gov, NCT00963352). All patients signed their informed consent.

### Computational work and statistical analysis

Exiqon GenEx qPCR analysis software was used to pre-process and manage the raw data generated from LightCycler 480. Normfinder identified the most stably expressed miRNAs in our samples to be miR-16-5p and miR-24-3p, and thus these two miRNAs were used as reference miRNAs. Quality control was performed to remove assays with a quantification cycle (Cq) value greater than 34. Further, relative quantities of the different miRNAs were calculated using the 2^−ΔΔCT^ method [50] and the expression in tumors were normalized to the expression in the ten normal samples. Finally, the expression level of each miRNA was transformed to logarithmic scale for further analysis. The R software version 3.3.2 “glmnet” package was used to perform the LASSO (Least Absolute Shrinkage and Selection Operator) in Cox mode to construct a miRNA classifier [51]. The prognostic value of the miRNA-signature was assessed using the 5-year disease free survival (DFS) which was defined as time from surgery to first recurrence or death of colon cancer if it occurred before documented recurrence. Based on the time to recurrence and the recurrence status, the X-tile plots (software version 3.6.1, Yale University School of Medicine, New Haven, CT, USA) were used to select optimum cut-points for each miRNA in the classifier in the study and the validation cohort [52].

The PI was calculated for each patient using the coefficients retrieved from the LASSO and the dichotomized expression value of each miRNA (0 or 1). The formula below was used where X_gi_ is the expression of the miRNAs in the signature for patient i, β_g_ is the LASSO coefficient for the target g.

$${\text{Prognostic Index}}_{i}=\overset{n}{\underset{g=1}{\sum }}\beta_{g}\times X_{\mathrm{gi}}$$

In order to dichotomize the PI for each patient into low and high risk of recurrence, receiver operating characteristic (ROC) curves were calculated to select the optimal cut-point value. The Kaplan-Meier method was used to calculate survival in patient groups with low- and high miRNA signature and p-values were based on the log rank test. To assess independence of the miRNA-signature as a prognostic marker in colon cancer, we used Cox-regression model and multivariate survival analyses. The statistical analyses were performed using SPSS (IBM SPSS Statistics, version 24).

MiRNA expression data from the TCGA-COAD project were downloaded from NIH National Cancer Institute Genomic Data Commons Data Portal (Supplementary Figure 2). Merged clinical information was retrieved from the FireBrowse-website (Broad Institute of MIT and Harvard, www.firebrowser.org). Counts for the mature miRNAs were normalized using trimmed mean of M (TMM) where counts from normal mucosa were used as reference samples. TMM is implemented in the R Bioconductor package edgeR [53].

## SUPPLEMENTARY MATERIALS FIGURES AND TABLES


