# Supplementary material for: Identification of a sixteen-microRNA signature as prognostic biomarker for stage II and III colon cancer
Source: Oncotarget. 2017 Sep 23;8(50):87837–47. doi: 10.18632/oncotarget.21237 (PMC5675676; doi:10.18632/oncotarget.21237)
Supplement: Supplementary file 1 [file oncotarget-08-87837-s001.pdf]

Identification of a sixteen-microRNA signature as prognostic biomarker for stage II and III colon cancer

SUPPLEMENTARY MATERIALS

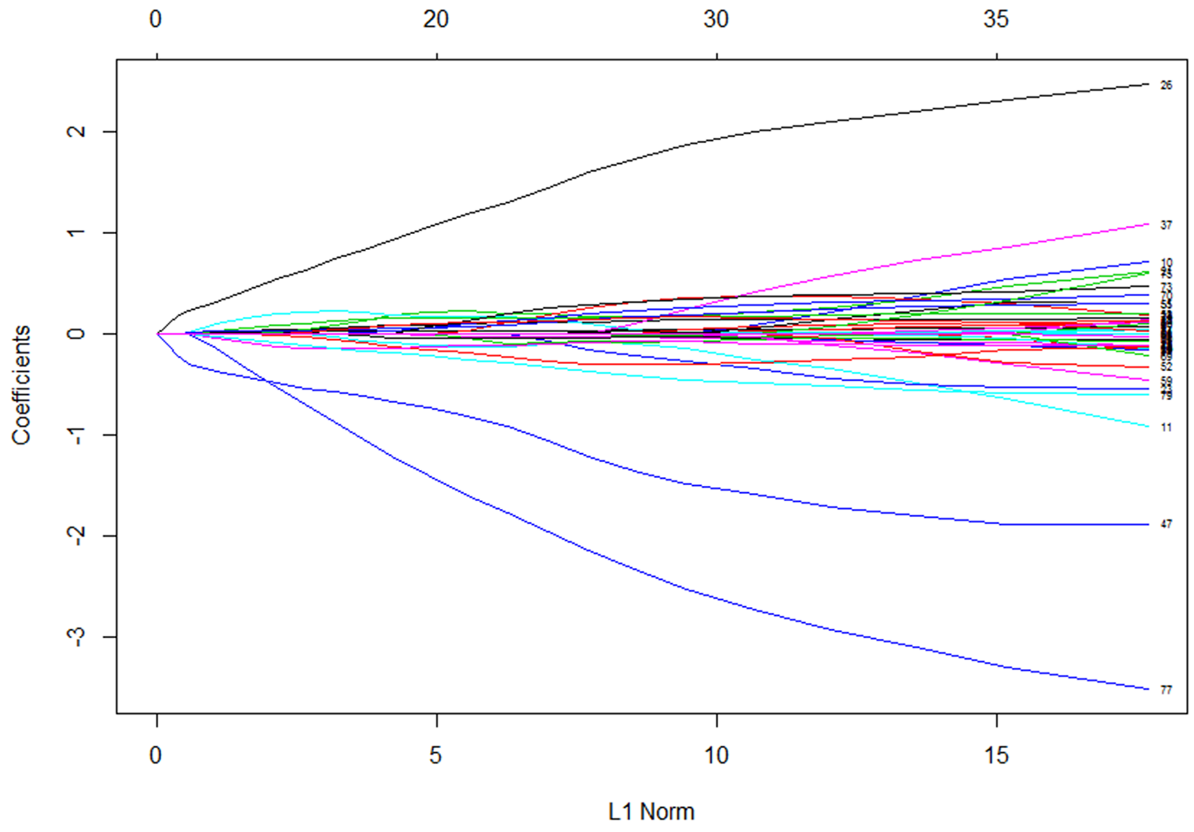

Supplementary Figure 1: The LASSO plot from LASSO regression analysis in the study cohort.

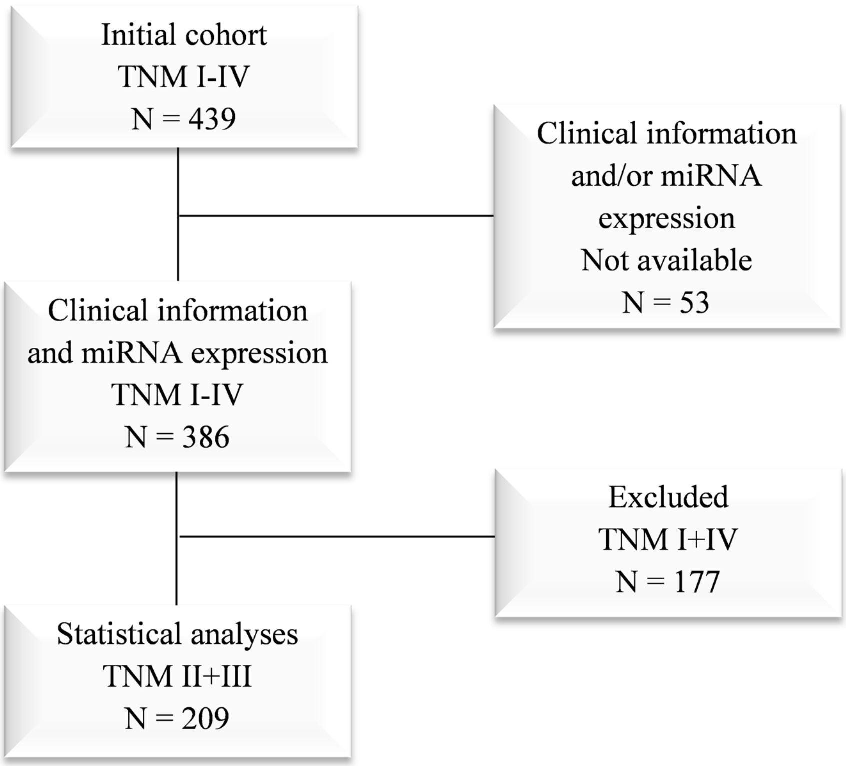

Supplementary Figure 2: Flowchart for the validation cohort (TCGA-COAD).

	1	2	3	4	5	6	7	8	9	10	11	12
A	hsa-miR-16-5p	hsa-miR-16-5p	hsa-miR-200a-3p	hsa-miR-200a-3p	hsa-miR-202-3p	hsa-miR-202-3p	hsa-miR-155-5p	hsa-miR-155-5p	hsa-miR-221-3p	hsa-miR-221-3p	hsa-miR-145-5p	hsa-miR-145-5p
B	hsa-miR-16-5p	hsa-miR-16-5p	hsa-miR-200a-3p	hsa-miR-200a-3p	hsa-miR-202-3p	hsa-miR-202-3p	hsa-miR-155-5p	hsa-miR-155-5p	hsa-miR-221-3p	hsa-miR-221-3p	hsa-miR-145-5p	hsa-miR-145-5p
C	UniSp3 IPC	UniSp3 IPC	hsa-miR-19a-3p	hsa-miR-19a-3p	hsa-miR-31-5p	hsa-miR-31-5p	hsa-let-7e-5p	hsa-let-7e-5p	hsa-miR-182-5p	hsa-miR-182-5p	hsa-miR-200b-3p	hsa-miR-200b-3p
D	UniSp3 IPC	UniSp3 IPC	hsa-miR-19a-3p	hsa-miR-19a-3p	hsa-miR-31-5p	hsa-miR-31-5p	hsa-let-7e-5p	hsa-let-7e-5p	hsa-miR-182-5p	hsa-miR-182-5p	hsa-miR-200b-3p	hsa-miR-200b-3p
E	hsa-miR-15b-5p	hsa-miR-15b-5p	hsa-miR-194-5p	hsa-miR-194-5p	hsa-miR-206	hsa-miR-206	hsa-miR-30b-5p	hsa-miR-30b-5p	hsa-miR-10a-5p	hsa-miR-10a-5p	hsa-miR-181b-5p	hsa-miR-181b-5p
F	hsa-miR-15b-5p	hsa-miR-15b-5p	hsa-miR-194-5p	hsa-miR-194-5p	hsa-miR-206	hsa-miR-206	hsa-miR-30b-5p	hsa-miR-30b-5p	hsa-miR-10a-5p	hsa-miR-10a-5p	hsa-miR-181b-5p	hsa-miR-181b-5p
G	hsa-miR-215-5p	hsa-miR-215-5p	hsa-miR-106a-5p	hsa-miR-106a-5p	hsa-miR-9-5p	hsa-miR-9-5p	hsa-miR-30d-5p	hsa-miR-30d-5p	hsa-miR-27b-3p	hsa-miR-27b-3p	hsa-miR-133a-3p	hsa-miR-133a-3p
H	hsa-miR-215-5p	hsa-miR-215-5p	hsa-miR-106a-5p	hsa-miR-106a-5p	hsa-miR-9-5p	hsa-miR-9-5p	hsa-miR-30d-5p	hsa-miR-30d-5p	hsa-miR-27b-3p	hsa-miR-27b-3p	hsa-miR-133a-3p	hsa-miR-133a-3p
I	hsa-let-7c-5p	hsa-let-7c-5p	hsa-miR-130a-3p	hsa-miR-130a-3p	hsa-miR-23a-3p	hsa-miR-23a-3p	hsa-miR-93-5p	hsa-miR-93-5p	hsa-miR-103a-3p	hsa-miR-103a-3p	hsa-miR-92b-3p	hsa-miR-92b-3p
J	hsa-let-7c-5p	hsa-let-7c-5p	hsa-miR-130a-3p	hsa-miR-130a-3p	hsa-miR-23a-3p	hsa-miR-23a-3p	hsa-miR-93-5p	hsa-miR-93-5p	hsa-miR-103a-3p	hsa-miR-103a-3p	hsa-miR-92b-3p	hsa-miR-92b-3p
K	Cel-miR-39-3p CP	Cel-miR-39-3p CP	hsa-miR-7-5p	hsa-miR-7-5p	hsa-miR-29a-3p	hsa-miR-29a-3p	hsa-miR-195-5p	hsa-miR-195-5p	hsa-miR-423-5p	hsa-miR-423-5p	hsa-miR-99a-5p	hsa-miR-99a-5p
L	Cel-miR-39-3p CP	Cel-miR-39-3p CP	hsa-miR-7-5p	hsa-miR-7-5p	hsa-miR-29a-3p	hsa-miR-29a-3p	hsa-miR-195-5p	hsa-miR-195-5p	hsa-miR-423-5p	hsa-miR-423-5p	hsa-miR-99a-5p	hsa-miR-99a-5p
M	hsa-miR-23b-3p	hsa-miR-23b-3p	hsa-let-7i-5p	hsa-let-7i-5p	hsa-miR-25-3p	hsa-miR-25-3p	hsa-let-7a-5p	hsa-let-7a-5p	hsa-miR-196a-5p	hsa-miR-196a-5p	hsa-miR-125b-5p	hsa-miR-125b-5p
N	hsa-miR-23b-3p	hsa-miR-23b-3p	hsa-let-7i-5p	hsa-let-7i-5p	hsa-miR-25-3p	hsa-miR-25-3p	hsa-let-7a-5p	hsa-let-7a-5p	hsa-miR-196a-5p	hsa-miR-196a-5p	hsa-miR-125b-5p	hsa-miR-125b-5p
O	hsa-miR-345-5p	hsa-miR-345-5p	hsa-miR-371a-5p	hsa-miR-371a-5p	hsa-miR-372-3p	hsa-miR-372-3p	hsa-miR-373-5p	hsa-miR-373-5p	hsa-miR-223-3p	hsa-miR-223-3p	UniSp3 IPC	UniSp3 IPC
P	hsa-miR-345-5p	hsa-miR-345-5p	hsa-miR-371a-5p	hsa-miR-371a-5p	hsa-miR-372-3p	hsa-miR-372-3p	hsa-miR-373-5p	hsa-miR-373-5p	hsa-miR-223-3p	hsa-miR-223-3p	UniSp3 IPC	UniSp3 IPC

	13	14	15	16	17	18	19	20	21	22	23	24
A	hsa-miR-19b-3p	hsa-miR-19b-3p	hsa-miR-20a-5p	hsa-miR-20a-5p	hsa-miR-26a-5p	hsa-miR-26a-5p	hsa-miR-27a-3p	hsa-miR-27a-3p	hsa-miR-186-5p	hsa-miR-186-5p	hsa-miR-126-3p	hsa-miR-126-3p
B	hsa-miR-19b-3p	hsa-miR-19b-3p	hsa-miR-20a-5p	hsa-miR-20a-5p	hsa-miR-26a-5p	hsa-miR-26a-5p	hsa-miR-27a-3p	hsa-miR-27a-3p	hsa-miR-186-5p	hsa-miR-186-5p	hsa-miR-126-3p	hsa-miR-126-3p
C	hsa-miR-143-3p	hsa-miR-143-3p	hsa-miR-149-3p	hsa-miR-149-3p	hsa-miR-106b-5p	hsa-miR-106b-5p	hsa-miR-181a-5p	hsa-miR-181a-5p	hsa-miR-1	hsa-miR-1	hsa-miR-141-3p	hsa-miR-141-3p
D	hsa-miR-143-3p	hsa-miR-143-3p	hsa-miR-149-3p	hsa-miR-149-3p	hsa-miR-106b-5p	hsa-miR-106b-5p	hsa-miR-181a-5p	hsa-miR-181a-5p	hsa-miR-1	hsa-miR-1	hsa-miR-141-3p	hsa-miR-141-3p
E	UniSp3 IPC	UniSp3 IPC	hsa-miR-22-3p	hsa-miR-22-3p	hsa-miR-18a-5p	hsa-miR-18a-5p	hsa-miR-200c-3p	hsa-miR-200c-3p	hsa-miR-29c-3p	hsa-miR-29c-3p	UniSp6 CP	UniSp6 CP
F	UniSp3 IPC	UniSp3 IPC	hsa-miR-22-3p	hsa-miR-22-3p	hsa-miR-18a-5p	hsa-miR-18a-5p	hsa-miR-200c-3p	hsa-miR-200c-3p	hsa-miR-29c-3p	hsa-miR-29c-3p	UniSp6 CP	UniSp6 CP
G	hsa-miR-34a-5p	hsa-miR-34a-5p	hsa-miR-146a-5p	hsa-miR-146a-5p	hsa-miR-26b-5p	hsa-miR-26b-5p	hsa-miR-107	hsa-miR-107	hsa-let-7b-5p	hsa-let-7b-5p	hsa-miR-214-3p	hsa-miR-214-3p
H	hsa-miR-34a-5p	hsa-miR-34a-5p	hsa-miR-146a-5p	hsa-miR-146a-5p	hsa-miR-26b-5p	hsa-miR-26b-5p	hsa-miR-107	hsa-miR-107	hsa-let-7b-5p	hsa-let-7b-5p	hsa-miR-214-3p	hsa-miR-214-3p
I	hsa-miR-29b-3p	hsa-miR-29b-3p	hsa-let-7g-5p	hsa-let-7g-5p	hsa-miR-150-5p	hsa-miR-150-5p	hsa-miR-17-5p	hsa-miR-17-5p	hsa-miR-210-3p	hsa-miR-210-3p	UniSp6 CP	UniSp6 CP
J	hsa-miR-29b-3p	hsa-miR-29b-3p	hsa-let-7g-5p	hsa-let-7g-5p	hsa-miR-150-5p	hsa-miR-150-5p	hsa-miR-17-5p	hsa-miR-17-5p	hsa-miR-210-3p	hsa-miR-210-3p	UniSp6 CP	UniSp6 CP
K	hsa-miR-205-5p	hsa-miR-205-5p	hsa-miR-100-5p	hsa-miR-100-5p	hsa-miR-15a-5p	hsa-miR-15a-5p	hsa-miR-101-3p	hsa-miR-101-3p	hsa-miR-191-5p	hsa-miR-191-5p	hsa-miR-192-5p	hsa-miR-192-5p
L	hsa-miR-205-5p	hsa-miR-205-5p	hsa-miR-100-5p	hsa-miR-100-5p	hsa-miR-15a-5p	hsa-miR-15a-5p	hsa-miR-101-3p	hsa-miR-101-3p	hsa-miR-191-5p	hsa-miR-191-5p	hsa-miR-192-5p	hsa-miR-192-5p
M	hsa-miR-148a-3p	hsa-miR-148a-3p	hsa-miR-132-3p	hsa-miR-132-3p	hsa-miR-222-3p	hsa-miR-222-3p	hsa-let-7d-5p	hsa-let-7d-5p	hsa-let-7f-5p	hsa-let-7f-5p	hsa-miR-30c-5p	hsa-miR-30c-5p
N	hsa-miR-148a-3p	hsa-miR-148a-3p	hsa-miR-132-3p	hsa-miR-132-3p	hsa-miR-222-3p	hsa-miR-222-3p	hsa-let-7d-5p	hsa-let-7d-5p	hsa-let-7f-5p	hsa-let-7f-5p	hsa-miR-30c-5p	hsa-miR-30c-5p
O	hsa-miR-20b-5p	hsa-miR-20b-5p	hsa-miR-24-3p	hsa-miR-24-3p	hsa-miR-10b-5p	hsa-miR-10b-5p	hsa-miR-21-5p	hsa-miR-21-5p	hsa-miR-92a-3p	hsa-miR-92a-3p		
P	hsa-miR-20b-5p	hsa-miR-20b-5p	hsa-miR-24-3p	hsa-miR-24-3p	hsa-miR-10b-5p	hsa-miR-10b-5p	hsa-miR-21-5p	hsa-miR-21-5p	hsa-miR-92a-3p	hsa-miR-92a-3p		

Supplementary Figure 3: Plate layout showing our Pick and Mix panel including the 84 miRNAs, inter-plate calibrators, and spike-in controls.
